# Adhesion molecules in multiple myeloma oncogenesis and targeted therapy

**DOI:** 10.2217/ijh-2021-0017

**Published:** 2022-04-26

**Authors:** Maroun Bou Zerdan, Lewis Nasr, Joseph Kassab, Ludovic Saba, Myriam Ghossein, Marita Yaghi, Barbara Dominguez, Chakra P Chaulagain

**Affiliations:** 1 Department of Hematology-Oncology, Myeloma & Amyloidosis Program, Maroone Cancer Center, Cleveland Clinic Florida, Weston, FL 33331, USA; 2 Saint-Joseph University, Faculty of Medicine, Beirut, Lebanon; 3 Department of Medicine & Medical Sciences, University of Balamand, Balamand, Lebanon

**Keywords:** bone marrow microenvironment, cellular adhesion molecules, drug resistance, monoclonal antibody, multiple myeloma, targeted therapies

## Abstract

Every day we march closer to finding the cure for multiple myeloma. The myeloma cells inflict their damage through specialized cellular meshwork and cytokines system. Implicit in these interactions are cellular adhesion molecules and their regulators which include but are not limited to integrins and syndecan-1/CD138, immunoglobulin superfamily cell adhesion molecules, such as CD44, cadherins such as N-cadherin, and selectins, such as E-selectin. Several adhesion molecules are respectively involved in myelomagenesis such as in the transition from the precursor disorder monoclonal gammopathy of undetermined significance to indolent asymptomatic multiple myeloma (smoldering myeloma) then to active multiple myeloma or primary plasma cell leukemia, and in the pathological manifestations of multiple myeloma.

**Practice points**

- Cell adhesion molecules (CAMs) are a subcategory of cell adhesion proteins located on the cell surface involved in cell–matrix interactions and cell–cell interactions.
- CAMs have been shown to play a vital role in the interactions between multiple myeloma (MM) cells and bone marrow (BM) cells; this interaction promotes proliferation and the survival of malignant plasma cells (MPC).
- MM cells can circulate, extravasate and then migrate back to the BM, a process mediated by numerous chemotactic factors and adhesion molecules.
- CAMs are strongly expressed by BM plasma cells (PC), but some adhesion molecules were found to be more significantly expressed in MPC in comparison to normal PC.
- Adhesion molecules like VLA-4 are involved in myeloma bone disease (MBD) through interactions with osteoblasts and osteoclasts which makes them interesting therapeutic targets in that case.
- CAM's expression is critical in the mechanisms underlying the adherence and detachment of PC from the bone marrow micro-environment (BMM), thus in the extramedullary spread of MPC; loss of CD56 expression on MPC is correlated with their capacity to disseminate and thrive outside of the BMM.
- MM remains incurable in most patients, with drug resistance driving relapse. Adhesion of MM cells to the BM stromal cells and the composition of the BM micro-environment contribute to MM drug resistance.
- CAMs play a role in the downregulation of the immune system by upregulating myeloid-derived suppressor cells and down-regulating dendritic cells and T cells.
- Daratumumab (a monoclonal anti-CD38 antibody), Lenalidomide, Bortezomib and Natalizumab are important examples of drugs interfering with CAMs that have been developed for the treatment of MM.

More than 40 years have passed since the first definitive demonstrations of molecular activity facilitating cell-cell adhesion [1–3]. Cell adhesion molecules (CAMs) are a subcategory of cell adhesion proteins located on the cell surface involved in binding with other cells or with the extracellular matrix (ECM) [4]. Adhesion plays an integral role in cell communication, regulation and development and maintenance of tissues. Some of the stimulating signals CAMs are involved with include but are not limited to cell differentiation, cell cycle maintenance, cell migration and cell survival. According to the cell adhesion model, the greater the number of chemical bonds a cell has on its surface the greater the cell-to-cell interaction. This model is also referred to as the point attachment model since it reflects the contact area between the cell and the surface to be a small, homogeneous region that mediates the initial attachment of the cell surface [5]. In addition to their function as a ‘molecular glue,’ CAMs are integral in affecting cellular mechanisms such as growth, contact inhibition and apoptosis. Changes in cell adhesion can be the defining event in a wide range of diseases including arthritis [6,7], atherosclerosis [8,9], osteoporosis [10,11] and cancer [12–14]. Cell adhesiveness is greatly reduced in malignancies. Reduced intercellular adhesiveness allows cancer cells to disobey social order which leads to the destruction of histologic architecture, a benchmark of malignant tumors [14]. In malignancies, variations in cell-matrix and cell–cell interactions are cell type- and oncogene-specific [8]. In multiple myeloma (MM), not only do CAMs regulate the interaction between MM cells and osteoblasts, osteoclasts, but they also mediate the interaction between MM and bone marrow stromal cells (BMSC), lymphocytes and endothelial cells. A deeper knowledge of CAMs can also play a role in stratifying patients, developing and employing targeted therapies, and in advancing research into understanding and overcoming drug resistance [15].

## Structure & families of CAMs

### Structure

**Figure 1.**
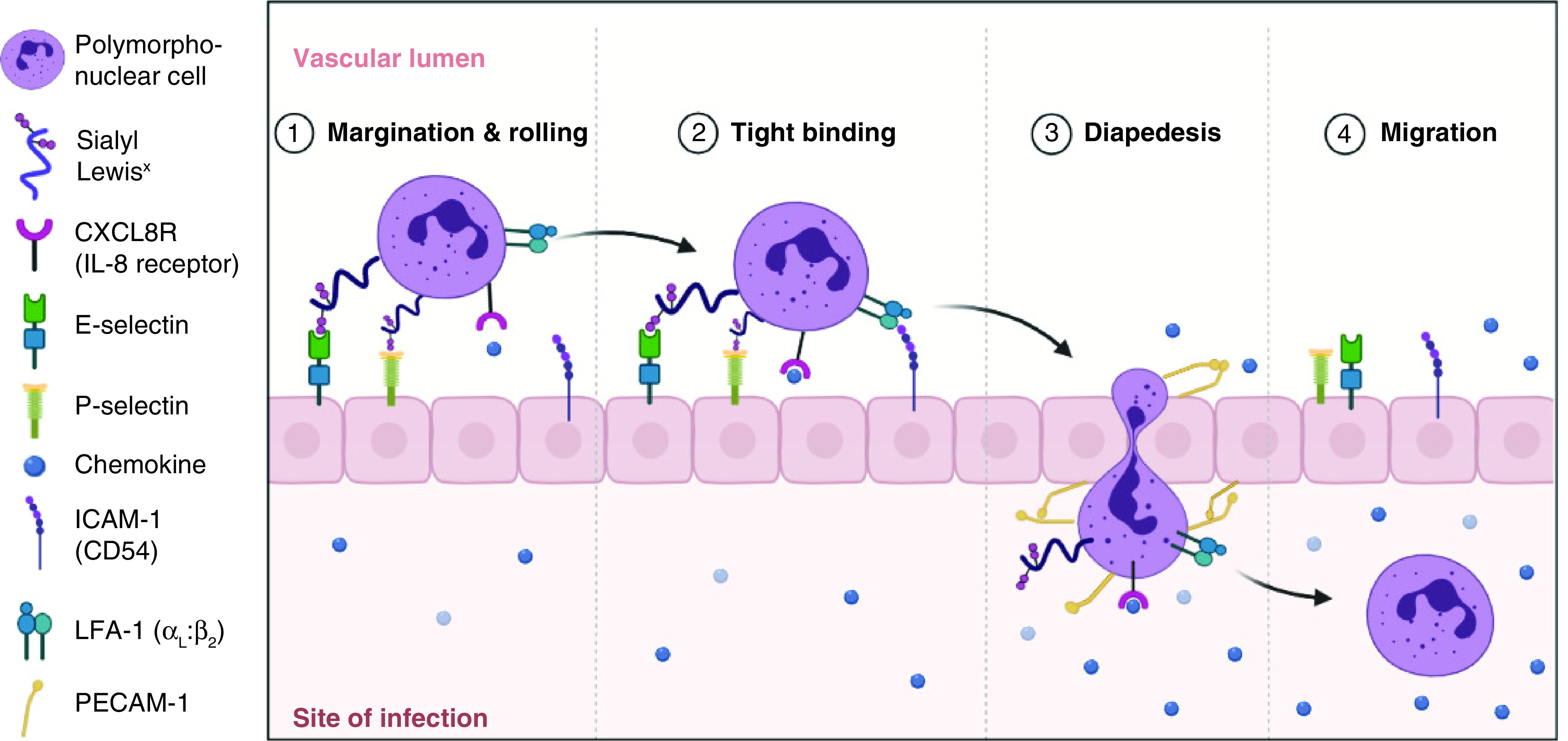
The process of leukocyte extravasation and the role of cell adhesion molecules.

Composed of three conserved domains, CAMs are typically single-pass transmembrane receptors [16,17]. The first domain represents an intracellular portion that interacts with the cytoskeleton, the second is a transmembrane domain, and the third is an extracellular portion. When CAMs bind with the same CAMs, the binding is known as homophilic binding. On the other hand, when cell adhesion occurs between molecules of two neighboring cells that are not identical, this binding is known as heterophilic binding. In some cases, a heterophilic binding can occur between CAMs and the ECM meshwork such as collagen fibers, fibronectin protein dimers and polysaccharides. CAMs have broad functions. One of which is depicted in Figure 1.

### Families of CAMs

There are four major superfamilies or groups of CAMs: Immunoglobulin super family CAMs (IgCAMs), cadherins, integrins and the superfamily of C-type lectin-like domains' proteins (CTLDs). According to Brackenbury *et al.*, three criteria have been utilized to distinguish among systems of embryonic cell adhesion: dependence on Ca^2+^, binding specificity, and the involvement of cell-surface molecules. CAMs can be divided into calcium-independent or calcium-dependent molecules [18]. Integrins and the IgCAMs belong to the latter while CTLDs and selectins belong to the former. The integrins play a role in cell–matrix interactions, while the rest of the CAMs participate in cell–cell interactions [16].

## CAMs in myelomagenesis

The proliferation of myeloma cancer cells and the acquisition of an invasive profile is related to integrin clustering. This is mediated by the rearrangement of cytoskeletal proteins and the recruitment of molecules involved in the signaling cascade, as well as the phosphorylation of pp60 Src and focal adhesion kinase [19]. The role of CAMs in MM pathophysiology is primarily related to the homing of tumor cells in the BM, which in turn aids in the recirculation of the malignant plasma cells (PC) and stimulates growth factor production [20]. Moreover, in patients with MM, drastic changes in the stroma of the BM have been observed. CAMs have been shown to play a vital role in the interactions between MM cells and BM cells; this interaction promotes proliferation and the survival of malignant plasma cells (MPC) [21,22]. Table 1 summarizes the expression of CAMs on normal cells compared with their expression on MM cells.

**Table 1.** Cell adhesion molecules phenotypic expression on normal versus myeloma plasma cells.

Plasma cell CAMs expressed	Normal plasma cell expression	Myeloma plasma cell expression
H-CAM (CD44)	Weak express +	Weak express +
VLA-4 (CD49d/CD29)	Weak expression +	Weak expression +
ICAM-1 (CD54)	Strong expression ++	Strong expression ++
N-CAM (CD56)	Weak/absent expression +/-	• Strong expression ++ • Absent expression in extramedullary involvement-
VLA-5- immature (CD49e/Cd29)^†^	Weak expression +	Absent/weak expression-/+
VLA-5+ mature (CD49e/CD29)^†^	Weak expression +	Absent/weak expression
CD38	Very strong expression +++	Very strong expression +++
CD19	Strong expression ++	Absent/weak expression-/+
LFA-1 (CD11a)	Strong expression ++	Weak expression +
CD40	Weak expression +	Weak expression +
LFA-3 (CD58)	Absent expression -	Strong expression ++
Syndecan	Strong presentation ++	Expression on small fraction
VLA-2 (CD49b)	Absent expression	Weak/moderate expression +/++
E-Selectin	Absent expression	Increased expression in progressing MM +++
CXCR4	Weak expression +	Strong expression ++
MAC-1	Absent expression	Strong expression ++
N-Cadherin	Strong expression ++	Strong expression in newly diagnosed patients ++
E- Cadherin	Strong expression +	Strong expression ++
PSGL-1	Strong expression ++	Very strong expression ++

† VLA-5 (CD49e/CD29) is expressed in cells which show a lower proliferative potential. It has a good potential for paraprotein product ion [20,23].

### Integrins

Integrins are heterodimeric membrane glycoproteins that are usually found on surfaces of diverse cell types. They act as the primary receptors that mediate the interaction between the extracellular matter and cell-cell adhesion molecules. Very late antigens such as the β1 integrin and in particular Very Late Appearing Antigen (VLA)-4 (CD49d) and VLA-5 (CD49e) have been prominent in MM cell lines and patient-derived MPCs [20]. The latter suggests that VLA-4 is one of the main integrins allowing MPCs to adhere to fibronectin through Arg-Gly-Asp dependent mechanisms in monoclonal gammopathy of undetermined significance [20,24]. Integrins are composed of α and β units. The α4 subunit was observed to be twice the level of β1 subunit in normal peripheral blood B lymphocytes which inherently express VLA-4. This suggests that the α4 subunits are not all necessarily functionally associated with the β1 subunit [20,25]. In contrast, MPCs express an equal amount of α4 and β1 subunits; inferring that MPC will possess a higher functional ability than normal peripheral blood B lymphocytes [20,23]. In addition, VLA-4 has been shown to play a role in facilitating migration and homing of MPCs to the BM stroma triggering an Interleukin (IL-6) secretion by the BM stroma [20,26–30]. VLA-5 (CD49e) expression by MPCs has been observed to vary as regards to fresh MPCs and human myeloma cell lines [20,31]. VLA-5 expression has been found to be upregulated upon contact with BM stroma [20]. A study by Kawano *et al.* recognized two subpopulations of MPCs in relation to VLA-5 expression. The first subpopulation is VLA-5 negative immature MPCs and the second subpopulation VLA-5+ mature MPCs [20,32]. VLA-negative immature MPCs have been observed to have a higher proliferative potential in addition to an increased responsiveness to IL-6, while VLA-5+ mature MPCs have a higher production of M protein [20,33].

The expression of other integrins in/by MPCs has also been reported. They include LFA-1, also known as lymphocyte function associated antigen, (CD11a/CD18) which was seen to be expressed on both human myeloma cell lines and MPCs. LFA-1 has been shown to be correlated with tumor cell growth [34]. In a study conducted by Ahsman *et al.*, LFA-1 expression was seen to be upregulated in fulminant disease states and to be scarcely expressed in patients with a stable disease. The latter suggests that LFA-1 expression correlates with plasma cell labeling index. Recent evidence proved that blocking LFA-1 by monoclonal antibodies can drastically reduce IL-6 production from the BM stroma. With that being said, LFA-1 may be implicated in important MPCs proliferation and cell adhesion interactions [20,35]. One possible explanation is probably through induction of IL-6 secretion by the BM stroma [33,35]. Similarly to VLA-4, LFA-1 is a ligand involved in B lymphocyte adhesion to dendritic cells (DCs) [20]. In a study conducted by Asosingh *et al.*, LFA-1 negative cells were not able to cause disease *in vivo*, but LFA-1 cells expressing 5T33MMvt were able to induce disease *in vivo* [21,36]. Drug resistance in MM patients was associated with MPCs expressing LFA-1 and VLA-4 [21,37]. MAC-1 (CD11b/CD18) which is also known as Membrane Attack Complex Type 1 has also been shown to be expressed on MPCs. MAC-1 mediates leukocyte adhesion to endothelial cell which in turn permits extravasation [20,24]. MAC-1 expression has been observed on MPCs, yet VLA-5 negative immature MPCs do not express MAC-1. Riet *et al.*, found a weak expression of VLA-6 which is a laminin receptor and CD51 which is a vitronectin receptor in malignant myeloma cells [23,38].

### Immunoglobulin superfamily CAMs

IgCAMs have different numbers of immunoglobulin domains. A large part of the IgCAMs members control cell behavior by acting as signal transducing receptors.

Pellat-Deceunynck *et al.* found that NCAM, neural cell adhesion molecule, (CD56) is strongly expressed on MPCs of patients with MM compared with normal PCs [20,38–44]. Research has suggested that the surface expression of NCAM in MPCs is controlled at the transcriptional level. This is due to the fact that some human myeloma cell lines have shown the absence on NCAM on their surface yet tested positive for the presence of its mRNA [20]. CD56 is an important marker because its increased expression promotes adhesion of neoplastic cells to osteoblastic cells [40]. A downregulation in the expression of NCAM, VLA-5 and CD11a with an increase in HCAM, homing cell adhesion molecule, (CD44) has been seen in patients with extramedullary spread of MPCs. The former features portray that NCAM could possibly aid in the localization of MM tumor cells to the BM stroma. HCAM may play a role in the passage of cells to the peripheral circulation [45,46]. Moreover, the down regulation of NCAM has been shown to facilitate the progression of MPC to extramedullary sites [20]. HCAM (CD44) and RHAMM, receptor for HA mediated motility, are hyaluronan receptors of MM cells [47]. HCAM (CD44) allows the interaction between malignant cells and the BM stroma, which increases IL-6 production and cancer proliferation. The use of monoclonal antibodies to CD44 has been shown to decrease IL-6 production by stromal cells, demonstrating the importance of the CD44/hyaluronate interaction [48].

MAC-1 is an integrin involved in leukocyte adhesion to the endothelium. It is not found in normal PCs, but rather in myeloma cells. Its functional role is not well understood [24]. LFA-3 (CD58) is expressed on MPCs while it is absent on normal PCs [20,32,41,44]. ICAM-1, Intracellular adhesion molecule-1, (CD54) is strongly expressed on MPCs, but it is also present on normal PCs. This highlights CD54 involvement in the physiological process of homing normal PCs to the BM [32,38,41,42,49,50]. Xu *et al.*, coined ALCAM, activated leukocyte cell adhesion molecule, (CD166) as a possible CAM which helps in malignant progression of MM [39]. CD166 is highly expressed in MM cell lines and primary BM cells from MM patients [39]. Moreover, myeloma cells homed more efficiently to the BM in immunodeficient mice expressing CD166 compared with those without it [39]. Last, a co-expression of ICAM-1 and LFA-1 was observed on 50% of the MPCs by Kawano *et al.*, and this was associated with the presence of *in vitro* homotypic aggregates [23,51].

### Cadherins

N-Cadherin plays an important role in regulating MM cell proliferation and ensuring its viability [52]. A study conducted by Sadler *et al.*, showed that when N-Cadherin is inhibited, MM cells were seen to increasingly proliferate; yet the addition of N-Cadherin antagonist peptide led to the death of MM adherent cells [52]. Studies conducted by Vandyke *et al.* and Groen *et al.*, showed that in 50% of newly diagnosed MM patients N-Cadherin expression is upregulated on PCs [53]. Moreover, the previously mentioned studies have shown that circulating N-Cadherin, which has a direct correlation with expressed cadherin on MPCs, are upregulated in 30% of newly diagnosed MM patients [53]. N-cadherin-mediated-cell-substrate or homotypic cell-cell adhesion has shown no contribution to myeloma growth *in vitro* [54]. In-vivo N-cadherin showed direct mediation of the bone marrow (BM) retention and localization of myeloma cells [54]. To add, N-cadherin *in vivo* facilitated an interaction between N-cadherin positive osteoblasts and myeloma cells [54]. Moreover, plasmacytoid dendritic cells (pDCs) have been shown to promote tumor growth and aid in immune suppression in MM [55]. When CpG was added to human pDCs they were activated and resulted in apoptosis of myeloma cells, yet direct contact of CpG with the myeloma cells resulted in the conversion of pDCs into tumor promoting cells [55]. E-Cadherin, which is expressed on myeloma cells and pDC cells was seen to help in the mediation of the interactions [55].

### Selectins

Selectins play a role in extravasation and homing of leukocytes to target organs. PSGL-1, p-selectin glycoprotein ligand-1, is the major ligand of P-selectin in neoplastic PCs and is expressed in high levels on normal and MPCs [56]. PSGL-1 was shown to have a role in the growth regulation, dissemination and contributed to the development of drug resistant in MM in relation to the bone marrow microenvironment (BMM) [57]. PSGL-1 is highly expressed on MM cells and regulates the homing and adhesion of MM cells to the microenvironment [57]. Moreover, activation of some integrins and downstream signaling was found to be regulated by PSGL-1. The latter interaction helps in regulation the proliferation of MM cell in the BMM and confers drug resistance [57].

E-Selectin has shown a role in homing and retention of MM cells in the BM [31,58]. Structures such as Sialyl Lewis A which are recognized by E-Selectin have been shown the help MM cells escape cytotoxic effects of bortezomib [58]. In a study conducted by Alexandrakis *et al.*, E-selectin was seen to be significantly increased with progression MM to advanced stages [59]. Moreover, a positive correlation was observed between IL-6, ICAM-1 and E-selectin [59]. The majority of MM patients relapse and become refractory to therapy since the tumor microenvironment plays a supportive role in development of drug resistance and spread [60]. A study which tested the effect of blocking E-selectin and CXCR4 using GMI-127 and GMI-1359 in order to sensitize MM cells to chemotherapy, found that decreasing the expression of E-selectin, and thus CXCR-4 mediated adhesion and MM cells chemotaxis, made the tumor cells more sensitive to treatment *in vivo* [60].

## Role of CAMs in MM

### CAMs in disease dissemination

Recent data has shown promising results in the role of CAMs for MM treatments, and possible cure. Understanding CAMs expression in MM at the molecular level is the first step toward devising a potential cure. MPCs have the ability to home in the bone BMM, supplementing myeloma cells with the optimal entourage for mediation of clonal proliferation and differentiation [20]. During the disease, the survival and proliferation of MM cells mainly rely on the BMM. The reciprocal interactions between the cancerous cells and the BM allow not only these cells' growth but also facilitate inhibition of apoptosis, angiogenesis and disease progression [61]. One of the particularities of MM is the presence of a significant amount of MPC within the BM. However, small amounts of MM cells can also be detected in the bloodstream called circulating plasma cells [61]. This implicates that MM cells can circulate, extravagate, and then migrate back to the BM. This multistep process is mediated by numerous chemotactic factors and adhesion molecules and implicates adhesion to the endothelium, followed by invasion of the subendothelial membrane and finally migration within the stroma [62]. At later stages of the disease, MM cells start to become stroma-independent and proliferate even in the absence of BMM due to the development of autocrine growth supporting loops [63,64]. Thus, an increasing number of extramedullary cells is observed, and extramedullary spread is common. This process is the result of molecular alterations involving adhesion molecules and chemokine receptor expression. The following summarizes the recent progress in the role of adhesion molecules in MM BM homing and extramedullary spread.

With the use of murine 5T MM model, Vanderkerken K *et al.* demonstrated that the specific localization of MM cells in the BM derives from a combination of selective homing and appropriate BMM [65]. Intravenously injected 5T2 MM cells (5T2MM) and 5T33 MM cells (5T33MM) migrate selectively to the BM, the liver, and the spleen. Interestingly, these cells only survive in the BM and the spleen [65,66]. This multistep homing process implies that appropriate cell surface receptors are found on MM cells allowing them to adhere and then traverse the endothelium as a first step [62]. To gain more insight into the subject, the expression of several adhesion molecules on human myeloma cell lines was analyzed [41,67–71]. These studies demonstrated that CAMs are strongly expressed by BM PC. The most relevant ones to note being the intercellular adhesion molecule ICAM-1 (CD54), the collagen-1 binding proteoglycan, the B cell differentiation antigen CD21, the lymphocyte homing receptor H-CAM (CD44), the fibronectin (FN) receptors called very late activation antigen-4 (VLA-4) (CD49d) and VLA-5 (CD49e), syndecan-1 (CD 138), and the plasma cell antigen MPC-1. Interestingly, a very similar PC phenotype was characterized in normal MM-free individuals, suggesting that these expressed adhesion molecules may also play a significant role in the normal BM homing process of PC. Nonetheless, some adhesion molecules were found to be more significantly expressed in MPC in comparison to normal PC. These include, the leukocyte adhesion molecule LFA-3 (CD58) [41] and the neural cell adhesion molecule N-CAM (CD56) [40]. Ahsmann EJM *et al.* also found that MPC can also express the lymphocyte function-associated antigen LFA-1 (CD11a/CD18) which was associated with tumor growth and homotypic tumor cell adhesion/aggregation [33]. The diverse expression of these different CAMs may suggest their direct implication in MPC homing and biology. However, to be biologically relevant, ligands of these receptors must be available within the tumor cell microenvironment. Therefore, Faid L *et al.* analyzed stromal layers of BM of MM patients and normal donors to find out which ligands are expressed. Results demonstrated that for most receptors found on the MPC surface, the corresponding ligands are actually found in the BM stroma [72]. As an example, it has been shown that MM cells strongly adhere to fibronectin using the very late activation antigen-4 (VLA-4) (CD49d) and to collagen-1 using syndecan-1 [71]. CD44 for its part, interacts with hyaluronic acid expressed on BM endothelium [73].

Furthermore, receptor-blocking antibodies against most of these CAMs (VLA-4, CD56, MPC-1, CD21) were found to block, but only partially, the adhesion of MM cells to the BM stroma, shedding the light on the presence of additional adhesion mechanism yet to be discovered [69,70]. Particular attention should also be given to the fibronectin receptor VLA-4. This CAM is functionally involved in the pre-B cell-stroma interactions which suggests a similar role in myeloma homing in the BM [27]. Moreover, it has been observed that VLA-4 – Fibronectin binding is an essential step that supports the IL6-mediated induction of PCs in normal BM since antibodies against VLA-4 were found to inhibit the secretion of IL6 in co-cultures of MM cells and stromal cells [27,35]. Finally, it is worth noting that some homing molecules could not be detected on myeloma PCs: the selectin molecule L-selectin and the collagen receptor VLA-2.

### Myeloma bone disease

MM is a hematologic malignancy associated with poorer overall survival than other B neoplasia. This is partly explained by the presence of myeloma bone disease (MBD). MBD affects about two-thirds of patients and is characterized by the occurrence of pathological bone events such as hypercalcemia, pain, pathological fractures, and spinal cord compression. This involvement of the skeletal system increases the morbidity and mortality of the disease, significantly altering the patients' quality of life in the process [74–80]. Adhesion molecules are involved in MBD through interactions with osteoblasts and osteoclasts. This makes adhesion molecules interesting therapeutic targets for the alleviation of bone damage associated with MM [81].

Osteoclastic cell precursors carry the RANK (receptor activator of nuclear factor kappa-B) receptor on their surface. The binding of RANKL (receptor activator of nuclear factor kappa-B ligand) expressed by osteoblasts is necessary for RANK-RANKL interaction to allow osteoclast differentiation and the initiation of bone resorption [82]. RANKL is not the only ligand of RANK. BMSC produce osteoprotegerin (OPG) which binds to RANK and inhibits osteoclast differentiation and thus protects against osteolysis. In multiple myeloma, there is an increase in RANKL and a decrease in OPG, Leading to a consequent increase in osteoclastic activity and promotes osteolytic bone lesions and pathological bone events [83]. It is thought that interactions between MPCs and stromal cells through adhesion molecules represent the process underlying these changes in multiple myeloma patients [84,85]. The integrin VLA-4 present on the surface of myeloma cells binds to VCAM-1, vascular cell adhesion molecule-1, and fibronectin, facilitating the proliferation of these tumor cells [86]. Antibodies to the alpha 4 portion of VLA-4 prevent binding of myeloma cells to fibronectin and intact stroma [87]. Osteoclastic activity is stimulated by VLA-4 interaction with VCAM-1. This binding leads to the production of MIP-1 alpha and MIP-1 beta by MM cells. These chemokines are responsible for an increase in RANK-L production leading to osteoclast activation and bone resorption [54]. VLA-4 plays a crucial role in the occurrence of MBD. This has been demonstrated in studies blocking VLA-4 adhesion where a decrease in osteolytic events was noted [87]. Notch-1, a single-pass transmembrane receptor, is strongly present on the surface of B-malignancy cells and interacts with the cell-surface protein, Jagged-1, of BMSC. This stimulates cell survival and growth [88,89]. The resultant stimulation of the Notch signaling pathway leads to an increase in osteoclastic activity. This results in an increase in the number and size of osteoclasts, potentiating their resorptive effect on bone, and leading to MBD [90]. Cadherins play a role in the localization of myeloma cells in the BM in the vicinity of N-cadherin expressing osteoblasts. The presence of this cadherin is associated with a worse prognosis [54]. N-cadherin binds Lrp5, which blocks the Wnt signaling pathway and thus decreases osteoblastic bone formation. This increases the number of osteoblasts expressing N-cadherin, and a vicious cycle of pathological bone degradation is initiated [91]. Xu *et al.* demonstrated in their work that the adhesion molecule CD166 leads to cancer progression and the occurrence of bone damage in patients with multiple myeloma. CD166 acts in a dual manner in the genesis of MBD. This is related to a decrease in osteoblastogenesis. The expression of CD166 by MM cells leads to a suppression of RUNX2 the primary transcription factor in the differentiation of mesenchymal stem cells into osteoblasts in the BM. CD166 also increases RANK-L, potentiating osteoclastic differentiation and osteolytic events [39].

### Extramedullary spread

During the course of MM, PC remains essentially located within the BM, and hardly ever spreads to other parts of the body, at the exception of the terminal stage of the disease during which patients develop spread at different extramedullary sites such as lung, liver, pleural fluid and ascites fluid [68,92]. Both the qualitative and quantitative expression of CAMs during the different stages of MM is critical in the mechanisms underlying the adherence and detachment of PC from the BMM [93]. Pellat-Deceunynck C *et al.* demonstrated that N-CAM (CD56) expression was normally seen on noncancerous BM PC, up-regulated on myeloma BM PC and, interestingly, down-regulated on PC of patients with extramedullary spread independently of their location (whether it was in the PB or in the BM). These findings seemingly indicate that the loss of CD56 expression on myeloma PC is correlated with their capacity to disseminate and thrive outside of the BMM. Furthermore, Pellat-Deceunynck C *et al.* also proved that osteoblastic cell lines interact with myeloma cells using CD56. Therefore, it is reasonable to say that the overexpression of CD56 on myeloma cells favors their adherence capacity within the BM while its downregulation favors the migration of myeloma cells in the PB [94]. On another note, many authors showed that the loss of VLA-4 (CD49d), VLA-5 (CD49e) and CD138 was statistically significant when comparing PC in disseminated disease and PC in BM localized disease [94–96]. Finally, Drucker L *et al.* and Tohami T *et al.*, recently demonstrated that the expression of tetraspanins, a family of ubiquitously expressed cell membrane proteins with many fundamental biological functions such as adhesion, migration and proliferation, also plays a role in the invasive capacity of MM cells [96,97]. Generally speaking, tetraspanins are usually down-regulated in malignant diseases and the metastatic potential of numerous cancers has been inversely linked to the expression of tetraspanins [98]. In the case of MM, CD81 and CD82 tetraspanins are down-regulated in PC especially during end stage disease [96]. Additionally, the induction of CD81 and CD82 overexpression in these PC decreased cell motility and invasion potential [97]. Therefore, it is likely that the deregulation of tetraspanin expression on MPC at later stages of the disease also plays a role in the dissemination of the neoplastic cells and thus the development of extramedullary spread. To sum up, it can clearly be affirmed that adhesion molecules showed lower expression on PC of patients with disseminated disease when compared with PC of patients with BM localized disease.

### Hypercoagulability in MM

Like all other cancers, MM is associated with a hypercoagulable state. There are several explanations for this. One explanation is related to adhesion molecules. Indeed, the endothelial damage induced by cancer cells or by chemotherapy causes an increase in the expression of adhesion molecules. This allows blood elements and tumor cells to adhere to the site of damage, leading to thrombosis through the secretion of thrombogenic substances [99]. IL-6 triggers the coagulation cascade and increases fibrinogen production. As previously mentioned, IL-6 production is partially mediated by adhesive interactions between malignant PCs and BM stromal cells [49,100].

## CAMs in myeloma drug resistance

Although there is constant development of new and improved therapeutics, MM remains incurable in most patients, with drug resistance driving relapse. There is a crucial need for the elucidation and resolution of intrinsic and acquired drug resistance pathways. These pathways vary widely and involve apoptosis pathways, DNA damage and repair mechanisms, modifications in gene expression, epigenetic events, and more [101].

Moreover, cell adhesion-mediated drug resistance is a well described phenomenon wherein adhesion of MM cells to the BM stromal cells and the composition of the BM micro-environment contribute to MM drug resistance. The adhesion can be to the BM stroma, hematopoietic niche constituents, endothelial cells, and the adjacent extra-cellular matrix [86]. This can reduce the sensitivity to chemotherapy agents, for instance, by upregulation of members of the anti-apoptotic Bcl-2 family and of ABC drug transporters, considered in the multidrug resistance proteins family, among other mechanisms [102].

As previously mentioned, the chemokine receptors CXCR4, CD49d (ITGA4) and CD44, highly expressed on PCs, are the main agents of adhesion of MM cells to the BM. CXCR4 has been linked to bortezomib resistance and poor patient outcomes with bortezomib. CXCR4 may be an interesting diagnostic biomarker of response to treatment. In addition, reduced CXCR4 expression was linked with extramedullary disease in murine specimens. CXCR4 mRNA expression was directly observed in bortezomib-resistant MM cell lines but not bortezomib-sensitive MM cell lines [103]. AMD3100 is a CXCR4 inhibitor that was shown to enhanced multiple therapeutic agents' action on MM cell lines *in vitro* by disrupting the adhesion to BM stromal cells in a murine myeloma model [104].

## Role of CAMs in Immune-microenvironment regulation

**Figure 2.**
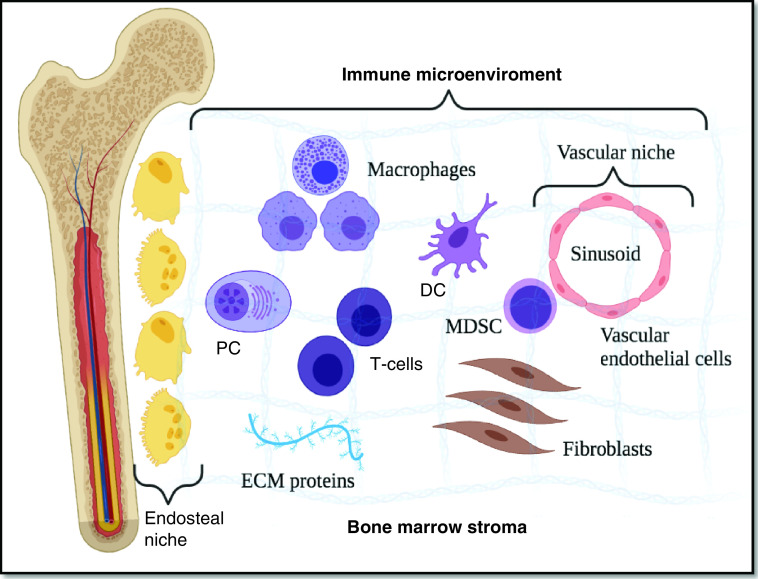
Overview of the bone marrow's immune microenvironment. The bone marrow microenvironment is divided into endosteal, and vascular niches set within a stroma of differentiated accessory or ‘stromal’ cells, such as fibroblasts, osteoclasts, osteoblasts, adipocytes, endothelial cells, macrophages and mast cells as well as ECM proteins. As for the term ‘immune microenvironment’, it refers to a functional compartment of differentiated immune cells located throughout the marrow stroma. DC: Dendritic cell; ECM: Extracellular matrix; MDSC: Myeloid-derived suppressor cell; PC: Plasma cell.

As previously mentioned, the neoplastic PC in MM vary from other B-cell malignancies by an almost exclusive homing to the BMM. The latter provides the adequate biological and physical support to induce clonal proliferation and differentiation. Anatomical and functional compartments, or niches, within the BM support different cellular populations, which together make up the BMM responsible for a sustained hematopoiesis. Classically, the BMM has been divided into endosteal and vascular niches set within a stroma of differentiated accessory or “stromal” cells, such as fibroblasts, osteoclasts, osteoblasts, adipocytes, endothelial cells, macrophages, and mast cells as well as ECM proteins consisting of a mixture of collagens, proteoglycans, and glycoproteins. As for the term ‘immune microenvironment’, it refers more to a functional compartment of differentiated immune cells located throughout the marrow stroma, rather than an anatomically distinct niche [105,106]. (Figure 2) Numerous fundamental changes and alterations in the BMM of MM patients are thought to promote growth of neoplastic progenitors. Focusing on the immune-microenvironment, it is now known that the evidence for the role of the immune system in the pathogenesis of MM is derived from a genetically humanized mouse model that demonstrated the microenvironment-dependent growth of malignant PCs [107]. The adhesion process occurring between MPC and both BM stromal cells and ECM proteins has been well-established. These adhesions can influence the growth, invasion, immune evasion and drug resistance of MM cells and are involved in lytic bone lesions and angiogenesis [102,108]. The following summarizes the recent progress in the role of adhesion molecules in MM tumor microenvironment and in particular, myeloma immune microenvironment.

### Adhesion molecules & T cells

T cells are crucial for the identification of tumor-specific antigens and for the killing of neoplastic cells [102]. CD8^+^ T cells are essential in protecting the host from malignant tumor growth [109]. High CD8^+^ T cell/Treg ratio in the tumor immune microenvironment has been associated with favorable prognosis in different human malignancies including MM [110,111]. Defects in T cell distribution and function have been reported in MM, including the decrease of CD4^+^ and CD8+T cell frequency, and abnormal Th1/Th2 ratio with impaired T cell responses [112]. As for immune checkpoint signaling it is upregulated in MM patients. Indeed, PD-1,programmed cell death protein-1, is overexpressed on T cells after activation and direct interaction with its ligand (PD-L1) on myeloma cells inhibits the T cell function by impairing proliferation and cytokine secretion [113]. Murine studies have shown that expression levels of integrin ligands on T cells directly impact T cell tumor infiltration. Fisher *et al.* demonstrated that ICAM-1 deficiency or blockade decreased CD8^+^ T cell infiltration into cancerous cells [114]. Furthermore, high expression of ICAM-1 and VCAM-1 on human studies, has been shown to correlate with higher density of CD8^+^ T cells also with prolonged disease free survival [115]. Also, Schmits *et al.* proved that LFA-1 deficiency in mice induced defects in CD8^+^ T cell and failure to reject immunogenic tumors [116]. In summary, integrins, integrin ligands and other cell adhesion molecules expressed on T cells mediate CD8^+^ T cell trafficking into tumors but more studies regarding their specific role in MM ought to be conducted.

### Adhesion molecules & DCs

DCs, act as professional antigen-presenting cells and as a link between innate and adaptive immunity [117]. DCs modulate immune responses and it is now widely accepted that DCs also play an important role in regulating the host immune responses to cancer [118]. Within the tumor microenvironment, many cancerous cells die naturally or because of anti-cancerous treatments. Consequently, DCs interact with dying tumor cells enabling them to acquire tumor antigens and amplifying immune response [119]. It is now presumed that MM patients' BM DCs are functionally defective. In fact, several authors already proved that some immunological properties – like the expression of HLA-DR, CD40, CD80 and CD86 - of DCs are altered during myelomagenesis, thus decreasing anti-tumor immune responses and leading to myeloma escape [120]. Adhesion receptors on DCs are involved in many of the processes involved in DC-mediated anti-tumor responses. Several receptors such as αVβ5-integrin expressed on immature DCs are involved in the interaction with and phagocytosis of dying cells. In addition, given that dying cells often become opsonized by complement component iC3b, DCs can also interact with dying tumor cells via the β2-integrins MAC-1 and αXβ2. However, β2-integrins often have anti-inflammatory effects in myeloid cells such as DCs, and these interactions lead to suppression of DC activation and, thus, tolerance [121]. Further, since inflammation of various levels has often been associated with tumor development, and ICAM-1 expression is up-regulated in lymphatic vessels during inflammation, interaction between MAC-1 and ICAM-1 expressed on DCs and inflamed lymphatic endothelium, respectively, may lead to decreased ability of DCs to activate T cells. Therefore, integrins on DCs may be involved in the uptake of dying tumor cells via adhesion receptors such as αVβ5-integrin, and in the subsequent initiation of DC-mediated anti-tumor responses. However, β2-integrins expressed on DCs may instead be involved in suppressing DC function [122]. In conclusion, although integrins play a key role in DC biology, the role of cell adhesion molecules in DC-mediated anti-tumor responses and DC-mediated tumorigenesis is still unclear and clearly requires further study, especially in human patients.

### Adhesion molecules & myeloid-derived suppressor cells

Myeloid-derived suppressor cells (MDSCs) are a diverse population of immature myeloid cells. They are known to be negative regulators in infectious processes, autoimmune diseases, and cancer [123]. Their role in the MM BM microenvironment is just starting to be untangled. MDSCs accumulate in the BM of MM patients and their increasing levels correlate with advanced disease stage and worsening prognosis [124]. Indeed, after reaching the tumor site, MDSCs successfully repress the anti-tumor immunity by numerous mechanisms such as by depleting T cell nutrients, by inducing the formation of reactive oxygen species and nitric oxide and by favoring the development of Tregs [125]. Focusing on CAMs, Jin *et al.* demonstrated that VLA-4, a highly expressed integrin in MM, is responsible for recruitment of MDSCs into the tumor site [126]. Later on, it was also found that tumor growth was significantly reduced in mice lacking activated form of VLA-4 [127]. Also, it is known that the integrin **MAC-1** is highly found on myeloid cells and plays an essential role in numerous myeloid cell functions [128]. In addition, Palmen MJ *et al.* showed that anti-MAC-1 monoclonal antibodies not only reduced myeloid cell tissue infiltration and inflammation but also decreased the recruitment of MDSCs into the tumor site [129]. Joined together, these studies prove that MDSCs act in favor of myeloma PCs and promote disease evolution. Therefore, MDSC-targeted therapies might overcome BM MM niche immunosuppression and increase the anti-tumor effect of additional therapies for MM patients.

### Adhesion molecules & tumor-associated vessels

The levels of CAMs such as ICAM-1, E-selectin, P-selectin, and T cell infiltration levels have also been shown to be correlated with malignancies such as in melanoma, glioblastoma, Merkel cell carcinoma and squamous cell carcinoma [130–133]. When squamous cell carcinoma samples were treated with a toll-like-receptor-7 agonist, imiquimod, up-regulated tumor vessels E-selectin expression was found on previously E-selectin negative tumor-associated vessels. This caused an increase in CLA+ CD8^+^ T cell influx into tumors, and a decrease in Treg frequency and tumor regression [134]. Integrins and VAP-1 on T cells and integrin ligands on endothelial cells are of crucial importance for T cell infiltration into tumors [132].

## CAM-targeted therapies in MM

### CD38

**Figure 3.**
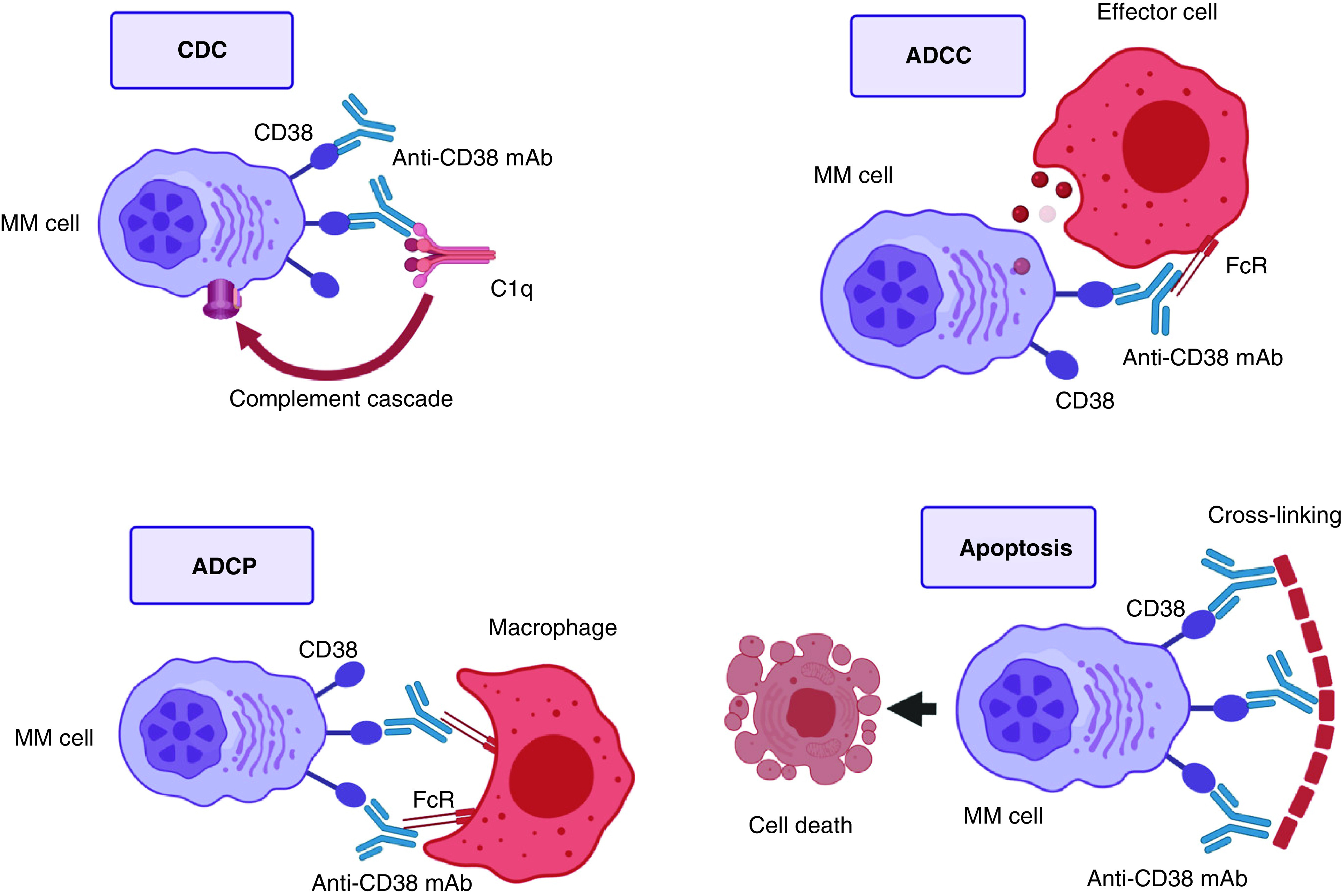
Anti-CD38 monoclonal antibodies mechanisms of action. **Top left:** mAbs bind CD38. The Fc fragment is bound by C1q, initiating the complement cascade, and resulting in a membrane attack complex, leading to cell lysis and death. **Top right:** mAbs bind CD38. The Fc fragment is then bound by an FcR-bearing effector cell, such as a natural killer cell, leading to activation of cytotoxic processes. **Bottom left:** mAbs bind CD38, and its Fc fragment is then bound by an FcR-bearing macrophage, inducing phagocytosis. **Bottom right:** FcR-mediated crosslinking of mAbs induces direct cellular apoptosis. ADCC: Antibody-dependent cell-mediated cytotoxicity; ADCP: Antibody-dependent cellular phagocytosis; CDC: Complement-dependent cytotoxicity; MAC: Membrane attack complex; MM: Multiple myeloma.

CD38 is a type II transmembrane glycoprotein of 46 kiloDaltons that is highly expressed on the surface of MM [135]. It functions as an ectoenzyme as well as a receptor involved in regulation of cell adhesion, cell migration, and signal transduction [136]. The very elevated cell surface density of CD38 on MM cells has made it a prime candidate for monoclonal antibody (mAb) targeting. Daratumumab (fully human; Janssen Pharmaceuticals) is the first therapeutic monoclonal anti-CD38 antibody approved by the Federal Drug Agency (FDA) for the treatment of MM both as a single agent and in combination with lenalidomide or bortezomib [137]. The mechanism of action of anti-CD38 mAb on MM cells is mediated by Fc-dependent immune mechanisms. Figure 3 illustrates the 4 main pathways of anti-CD38 action: complement-dependent cytotoxicity (CDC), antibody-dependent cellular cytotoxicity (ADCC), antibody-dependent cellular phagocytosis (ADCP) and direct apoptosis after secondary cross-linking [138].

### Lenalidomide

Lenalidomide (and its predecessor Thalidomide), a potent immunomodulatory (IMiDs), is highly active agents in the treatment of MM [139]. IMiDs amplify the cytotoxic T-lymphocyte and natural killer (NK) activity against multiple myeloma cells [140]. Nonetheless, in addition to these immunomodulatory effects, recent studies have indicated that its mechanisms of action are highly complex and involve diverse molecular targets in the BM microenvironment. Studies showed that lenalidomide/thalidomide decrease gelatinase, matrix metalloproteinase 2 and 9, production by interfering with multiple integrin-mediated signaling pathways involved in the regulation of cell motility, invasion and survival [141]. Furthermore, treatment with lenalidomide decreases v3-integrin in tartrate-resistant acid phosphatase positive cells, attenuates RANKL secretion in bone marrow stromal cells (BMSCs) [142]. This IMiDs-mediated disruption of MM-BMSCs signaling may then disrupt multiple contact-dependent tumor cell interactions with the bone marrow microenvironment which are crucial for the survival of MM cells.

### Bortezomib

Similar to IMiDs, the proteasome inhibitor bortezomib also has its place in the treatment of patients with newly diagnosed or relapsed MM [143]. Treatment of MM cells with bortezomib leads to the induction of apoptosis by interfering with NF-KB signaling [144], leading to the accumulation of misfolded proteins within ER with cellular death [145]. Interestingly, in addition to these mechanisms, proteasome inhibition downregulates the expression of a4b1 and a5b3 integrins, important mediators of the MM-BMSCs signaling, which enhances the survival of MM cells [146].

### Natalizumab

In contrast to the IMiDs and proteasome inhibitors which indirectly affect MM cells adhesion properties, many classes of drugs are directly aimed at disrupting the myeloma cell-stromal cell interaction and may have a promising role in the management of MM. To date, targeting of integrins in multiple myeloma have mainly focused on VLA-4. A monoclonal antibody (PS/2), which binds to the a4 chain of murine integrin VLA-4, showed important anti-MM activity in mice injected with 5TGM1 MM cells as well as significant decrease in tumor burden in the bone marrow, spleen, liver, of treated animals [147]. Natalizumab, a recombinant humanized IgG4 monoclonal antibody that binds integrin-a4, has demonstrated ability to inhibit adhesion of MM cells to noncellular and cellular components of the BM microenvironment [148].

### Other

N-cadherin antagonist peptide (ADH-1) has shown to act by increasing the permeability of tumor-associated vasculature and hence leading to an increase in therapeutic drug delivery, which subsequently leads to an enhanced response [149]. LCRF-006 is a mimetic of the classical cadherin His-Ala-Val region of ADH-1 [149]. It was found that LCRF-0006 worked by disrupting in a rapid transient and reversible manner the endothelial cell junctions which in turn lead to the increase in the vascular permeability at sited of Multiple Myeloma tumor *in vivo* [149]. It was also found that LCRF-0006 increased the *in vivo* anti-MM tumor response to low dose bortezomib [149].

Table 2 depicts some of the agents discussed in this section along with others.

**Table 2.** Mechanism of actions of various molecules in multiple myeloma.

Drugs	Mechanism of action
**Non-mAb**	
Thalidomide^†^, Lenalidomide^†^, Pomalidomide^†^, Iberdomide	Immunomodulator
Panobinostat^†^, Vorinostat, Abexinostat, Belinostat, Givinostat, Romidepsin, Entinostat, Tacedinaline	Histone deacetylase inhibitors
Sotatercept	Activin inhibitor
Evofosfamide	Hypoxia-activated prodrug
Plerixafor	CXCR4 antagonist
Anti-CD19, anti-CD138, anti-BCMA, anti-SLAM7 CAR—T cells	Chimeric antigen receptor (CAR) T cells
Venetoclax	BCL-2 inhibitor
DANFIN	NF-κB inhibitor
Sorafenib, Vemurafenib, Cobimetinib, Selumetinib	RAS/RAF/MEK/ERK inhibitors
Palbociclib	CDK4/6 inhibitor
Dovitinib, BGJ398, MFGR1877S, AZD4547	FGFR inhibitors
Clioquinol, SC-06, BEZ235, BAY80-6946, MK-2206	PI3K/AKT/mTOR inhibitors
Selinexor^†^	Selective inhibitor of nuclear export
**mAb**	
Blinatumomab, AMG 701, REGN5458	Bispecific T cell engagers
Ulocuplumab	Anti-CXCR4 mAb
Nivolumab, Pembrolizumab, Cemiplimab, Cetrelimab	Anti-PD-1 mAbs
Atezolizumab, Avelumab, Pidilizumab	Anti-PD-L1 mAbs
Ipilimumab	Anti-CTLA-4 mAb
Alemtuzumab	Anti-CD52 mAb
Siltuximab	Anti-IL-6 mAb
BI-505	Anti-ICAM-1 mAb
Daclizumab	Anti-CD25 mAb
AVE1642	Anti-IGF1R mAb
BHQ880	Anti-DKK1 mAb
Bevacizumab	Anti-VEGF mAb
Tabalumab	Anti-BAFF mAb
Daratumumab^†^, Isatuximab^†^	Anti-CD38 mAb
Elotuzumab^†^	Anti-SLAMF7 mAb
Denosumab^†^	Anti-RANKL mAb
Belantamab mafodotin^†^	Anti-BCMA conjugated mAb

† mAb-approved drugs.

mAb: monoclonal antibody.

## Conclusion

Progress in our comprehension of the pathophysiology of MM and the advent of novel and targeted therapies have heralded a remarkable decade of progress in myeloma. This has in turn translated into better patient outcomes. Yet, despite these advances, MM remains an incurable hematologic malignancy. CAMs have emerged as important players in this disease and their domain structures, superfamily groupings, and interactions have been heavily studied. CAMs play a remarkable role in the development of targeted therapies, the understanding of MM pathogenesis/dissemination and the emergence of drug resistance. They are involved in numerous cellular mechanisms such as notably epigenetic stability, BM homing, immune microenvironment regulation and more. CAMs have been shown to play a vital role in the interactions between MM cells and BM cells, promoting proliferation and survival of the former. Some involved mechanisms include but are not limited to, inhibition of apoptosis, induction of angiogenesis and alteration of cell–cell interaction. Clinically, this translates into myeloma bone disease – mainly VLA-4 mediated, extramedullary spread – mainly by N-CAM (CD56) downregulation, and the emergence of drug resistance, whether directly or indirectly. The quasi-omnipresence of CAMs has allowed their use as biomarkers of disease as well as targets for therapies that will push the boundaries of the care available to MM patients. Actual treatments of note interacting with CAMs include anti-CD38 monoclonal antibodies (ie. daratumumab), immunomodulators IMiDs (i.e., lenalidomide), proteasome inhibitors downregulating a4b1 and a5b3 integrins (ie. Bortezomib) and a4-VLA-4 integrin inhibitors (ie. Natalizumab). Anti-SLAMF7 drug, CAR T cells, and anti-BCMA drugs are also emerging treatment options. Finally, it is worth noting that the successful incorporation of CAM-targeting drugs into the clinical arsenal in MM may pave the way to their use in other hematological malignancies.

## Future perspective

Whether it is liquid biopsies, immunotherapy, CAR T cells or FoundationOne, the field of cancer therapy has been revolutionized during the last decade. CAMs seem to be promising targets for cancer therapy. Clinical trials directly targeting integrins on malignant cells have not been promising so far. A more efficient approach to consider may be to increase the expression or function of β_2_-integrins on immune cells or their ligands on tumor-associated blood vessels. This would enhance anti-tumor responses in a more efficient approach. Enhancing β_2_-integrin function could open a chance to overcome immunotherapy's inability to access the tumor microenvironment. In addition, β_2_-integrins functions and expression regulation needs to be addressed so that harmful-treatment related adverse events are avoided.
